# Molecular identification of genus *Duttaphrynus* from Punjab, Pakistan

**DOI:** 10.1080/23802359.2020.1810143

**Published:** 2020-08-26

**Authors:** Saddam Hussain, Syed Mohsin Bukhari, Arshad Javid, Ali Hussain, Muhammad Rashid, Waqas Ali

**Affiliations:** a Department of Wildlife and Ecology, University of Veterinary and Animal Sciences, Lahore, Pakistan; b Faculty of Fisheries and Wildlife, University of Veterinary and Animal Sciences, Lahore, Pakistan

**Keywords:** *Duttaphrynus stomaticus*, *Duttaphrynus melanostictus*, *Bufo surdus*, *Bufo viridis* and *Bufo*

## Abstract

The Indus valley toad and common Asian toad are widely distributed toads in Pakistan. There is doubt in the taxonomic position of species within the genus *Duttaphrynus* in Pakistan as most of the species identified on morphology. Previously, *Bufo melanostictus hazarensis* identified on morphology but during the present study, it is confirmed as *Duttaphrynus melanostictus-*based COI sequences (MK941836). The interspecific divergence between *Duttaphrynus stomaticus* and *D. melanostictus* was 16%. The intraspecific divergence of *D. stomaticus* (MK947909.1) was ranging from 0% to 1% while the intraspecific divergence of *D. melanostictus* (MK941836) was high ranging from 10% to 11%. Overall, genetic variation between the species of genus *Duttaphrynus* based on p-distance was 14%. In our recommendation, a large-scale molecular identification of amphibians should take into consideration for exact species identification to report any new species from Pakistan.

## Introduction

Global decline in amphibian’s species is well reported in the literature and different factors including disease, pollution, predation, acid rain, global warming, habitat loss, and anthropogenic hazards are main causes. Overall, 30% amphibians of the globe are threatened within extinction (Ali et al. 2018). Morphological keys are considered authentic to identify amphibians but molecular identification should also take into consideration for exact species identification to resolve cryptic species ambiguities (Becker et al. 2007; Ali et al. 2017).

Molecular identification is a significant tool and has been useful to study genetic diversity in a number of species. Recent studies indicated that mtDNA genes, such as Cytb and COI genes are effective for cryptic species identification (Vences et al. 2005).

In tropical regions, there are many endangered as well as many cryptic amphibians species that are yet to be identified. The taxonomic position of many amphibians is still unclear in Pakistan as very few molecular studies carried out so far (Ali et al. 2020). In this context, the present study was planned to identify species of Genus *Duttaphrynus* from Punjab, Pakistan.

## Materials and methods

### Sampling

Sampling was done from selected sites of district Bahawalangar, Pakistan. One specimen of each species was preserved in 75% alcohol for molecular characterization. The voucher specimens (ZMUVAS14 & ZMUVAS7) were placed at Zoological Museum, UVAS, Pakistan.

### Molecular identification

Each sample was identified using identification keys (Khan 2006) and morphometric measurements were noted following (Ali et al. 2016, 2020).

### DNA sequencing

Genomic DNA was extracted from preserved tissues through the salt extraction method. Purity of DNA was checked through 1% agarose gel. DNA was amplified using primer set LCO1490 (5′-GGTCAACAAATCATAAAGATATTGG-3′) and HCO2198 (5′-TAAACTTCAGGGTGACCAAAAAATCA-3′) (Vieites et al. 2009). PCR amplification was done in 25 µl volume using 6 µl double distilled water, 1 µL (25 mM) primer F, 1 µL (25 mM) primer R, 12 µL master mix and 5 µl of DNA. The PCR amplification consisted of 3 min denaturing at 94 °C followed by 40 cycles of 94 °C for 30 s, primer annealing for 30 sec at 42 °C and elongation for 1 minute at 72 °C, with final 10 min at 72 °C and infinity at 4 °C. PCR products were checked through 1.2% agarose gels. All the samples were Sanger sequenced on ABI3730XL DNA Analyzer (Applied Biosystems) from Korea.

### Phylogenetic analysis

The obtained DNA sequences were analyzed using Bioedit 7.0 and aligned using Clustal X. The DNA sequences were subjected to BLAST analysis. Closely related sequences from the GenBank were obtained and incorporated in the neighbor-joining (NJ) tree analysis. Neighbor-joining tree was constructed using 100 bootstrap replicates in MEGA 10. Genetic variations between and within species were calculated based on p-distance.

## Results

During the present study, five specimens of *Duttaphrynus stomaticus* and *Duttaphrynus melanostictus* were captured from the study area for morphological parameters. One specimen of each species was preserved in 75% alcohol for DNA sequencing.

### Taxonomic position

Amphibia, Linnaeus, 1758

Anura Fischer von Waldheim, 1813

Bufonidae Gray, 1825

*Duttaphrynus stomaticus* Lütken, 1864

*Duttaphrynus melanostictus* (Schneider, 1799)

### Morphology

*Duttaphrynus stomaticus* commonly known as Indus valley toad and is widely distributed across Pakistan. *Duttaphrynus stomaticus* does not have cranial crests, tympanum is distinct and round, 1st and 2nd finger is subequal, the parotid gland is longer than broad. The snout to vent length was 48.81 ± 1.19 mm, the head length was 11.54 ± 1.17 mm and the weight was 8.40 ± 1.13 g (*n* = 5).

*Duttaphrynus melanostictus* are commonly known as Asian toad and have scattered distribution in Pakistan. *Duttaphrynus melanostictus* are stout, medium-sized toad and have short limbs. The skin is dry and thick, prominent cranial ridges and expanded parotid gland. The tympanum is oval shape and prominent. The snout to vent length was 49.10 ± 0.90 mm, head length was 12.14 ± 0.27 mm and weight was 9.18 ± 1.7 g (*n* = 5).

### Coloration

*Duttaphrynus stomaticus* have dorsum light gray or olive to almost black, gray to dark reticulation, ventral side is dirty white and tip of digits dark brown. *Duttaphrynus melanostictus* is mostly pale yellow-brown in color with dark brown spots. The back possessed warts and is encircled with black or dark pigments.

### Natural history

Both species are nocturnal and hide under rocks and leaf litter and logs during day time. The toads are slow moving and feeding on insect which is considered as pests to human. Breeding depends on water in areas having pronounced rainy and dry seasons. Mating is started typically at the beginning of the monsoon. Eggs are usually deposited in water ponds.

### Phylogenetic relationship

The DNA sequences of *D. stomaticus* and *D. melanostictus* were submitted to GenBank and accession numbers were obtained (MK947909.1 and MK941836). Overall, Pakistan is represented by 12 toad species, namely *Bufo himalayanus, Bufo latastii, Bufo melanostictus hazarensis, Bufo olivaceous, Bufo pseudoraddei, B. pseudoraddei baturae, Bufo baturae, Bufo siacheninsis, Bufo stomaticus, B. surdus, Bufo viridis* and *Bufo viridis zugmayri.*

**Figure 1.**
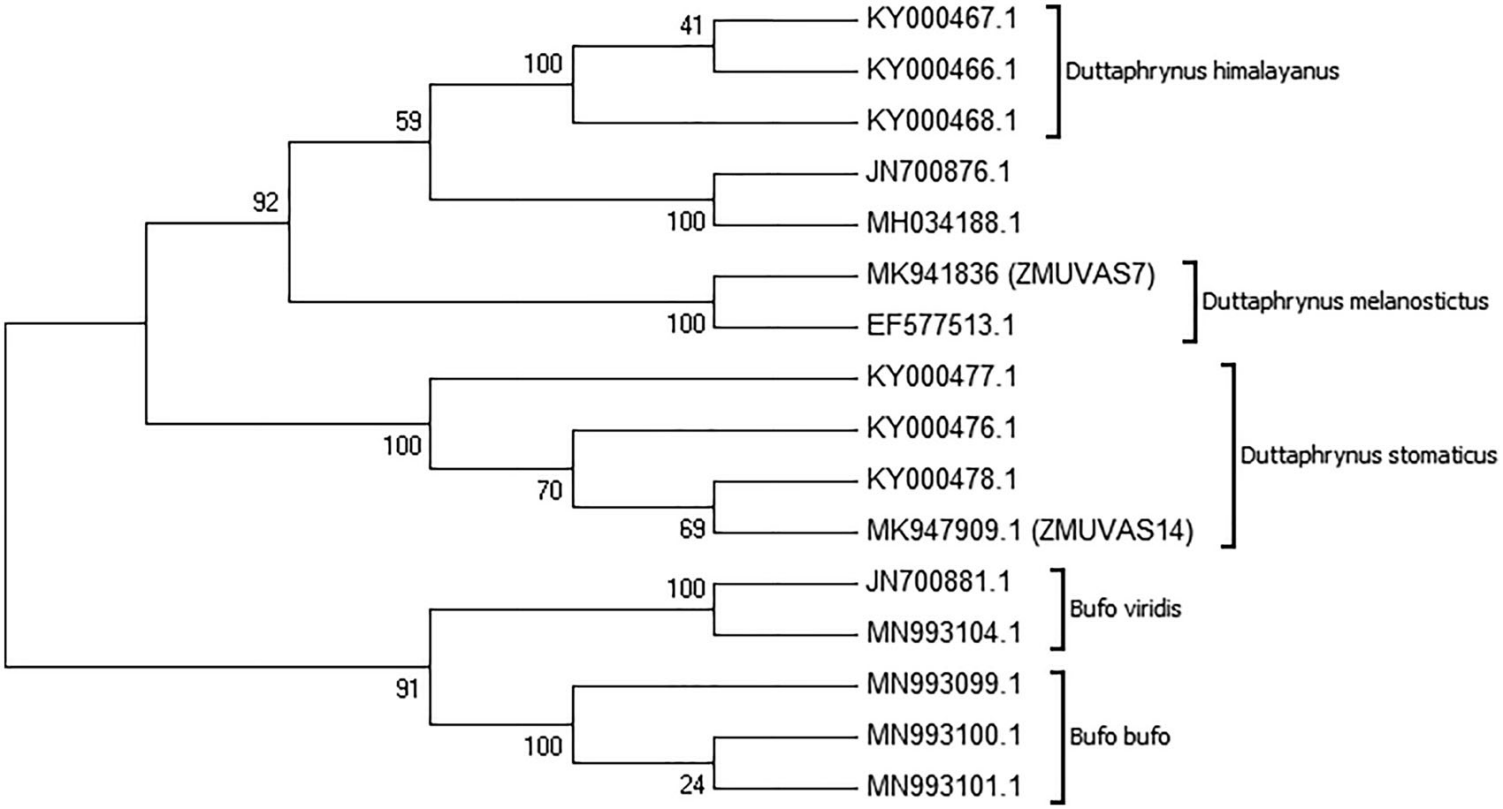
Phylogenetic relationship of genus *Duttaphrynus* using the neighbor-joining tree based on p-distance. Numbers on branches represent bootstrap values.

DNA sequences of *B. latastii, B. melanostictus hazarensis, B. olivaceous, B. pseudoraddei, B. pseudoraddei baturae, B. baturae, B. siacheninsis, Bufo surdus* and *B. viridis zugmayri* are not available in GenBank to validate their taxonomic position in Pakistan. The retrieved sequences of *D. stomaticus, D. melanostictus, Duttaphrynus himalayanus, Bufo bufo* and *B. viridis* from GenBank incorporated in Neighbor-joining tree analysis (Figure 1).

After trimming the bases, the obtained COI fragments were 660 bp aligned with NCBI sequences consist of 630 bp. The interspecific divergence between *D. stomaticus* and *D. melanostictus* was 16%. The intraspecific divergence of *D. stomaticus* was ranging from 0% to 1% while the intraspecific divergence of *D. melanostictus* was high ranging from 10% to 11%. Overall, genetic variation between the species of genus *Duttaphrynus* was 14% based on p-distance (Table 1).

**Table 1.** Genetic variation between the species of genus *Duttaphrynus* from Punjab based on p-distance.

Species	*Duttaphrynus stomaticus*				*Duttaphrynus melanostictus*		*Duttaphrynus himalayanus*			*Duttaphrynus melanostictus*		*Bufo viridis*		*Bufo*		
*Duttaphrynus stomaticus*	KY000478.1															
	KY000476.1	0.004														
	MK947909.1	0.002	0.002													
	KY000477.1	0.013	0.009	0.011												
*Duttaphrynus melanostictus*	JN700876.1	0.132	0.132	0.130	0.135											
	MH034188.1	0.132	0.132	0.130	0.135	0.000										
*Duttaphrynus himalayanus*	KY000468.1	0.173	0.171	0.171	0.175	0.115	0.115									
	KY000467.1	0.173	0.171	0.171	0.175	0.115	0.115	0.000								
	KY000466.1	0.173	0.171	0.171	0.175	0.115	0.115	0.000	0.000							
*Duttaphrynus melanostictus*	MK941836.1	0.154	0.152	0.152	0.156	0.115	0.115	0.132	0.132	0.132						
	EF577513.1	0.139	0.137	0.137	0.141	0.107	0.107	0.132	0.132	0.132	0.021					
*Bufo viridis*	JN700881.1	0.156	0.154	0.154	0.158	0.162	0.162	0.179	0.179	0.179	0.156	0.154				
	MN993104.1	0.160	0.158	0.158	0.162	0.154	0.154	0.175	0.175	0.175	0.158	0.156	0.049			
*Bufo*	MN993099.1	0.173	0.171	0.171	0.173	0.188	0.188	0.179	0.179	0.179	0.190	0.186	0.152	0.160		
	MN993100.1	0.173	0.171	0.171	0.173	0.188	0.188	0.179	0.179	0.179	0.190	0.186	0.152	0.160	0.000	
	MN993101.1	0.173	0.171	0.171	0.173	0.188	0.188	0.179	0.179	0.179	0.190	0.186	0.152	0.160	0.000	0.000

## Discussion

In the recent past, amphibians have taken significant attention of the scientific community because of widespread declines in species caused by many anthropogenic activities and climate change. Overall, 43% of amphibians facing decline and 32.5% species globally threatened (Howlader 2011).

Pakistan is represented by 24 species and out of these 9 species are endemic to the country. In Pakistan, only a few molecular studies have been carried to identify amphibians species. Recently, *Euphlyctis kalasgramensis* reported from first from Pakistan based on 16S rRNA sequence (Ali et al. 2020).

Morphological identification of amphibians species might not be enough and can cause an enormous underestimation of diversity (Vieites et al. 2009). In amphibians, robust molecular divergence is not always based on morphological variations; hence, genetic data can provide key evidence on population pattern and detection of cryptic species (Vieites et al. 2009). Khan (2006) reported *D. melanostictus* as *Bufo melanostictus hazarensis* based on morphological parameters. The results of the present study suggested the existence of two distinct taxonomic species from study area.

## Conclusions and recommendation

Previously, *Bufo melanostictus hazarensis* identified on the basis of morphology by Khan (2006) but during the present study, it is confirmed as *D. melanostictus-*based COI sequences. There is ambiguity in the taxonomic position of many species within the genus *Duttaphrynus* in Pakistan as many resembling species have been identified as separate species on the basis of morphological parameters. In our recommendation, a large-scale molecular identification of amphibians should take into consideration for exact species identification and to report any new species from Pakistan.
